# Structural insights into antigen recognition of an anti-β-(1,6)-β-(1,3)-D-glucan antibody

**DOI:** 10.1038/s41598-018-31961-x

**Published:** 2018-09-12

**Authors:** Kwang Hoon Sung, Jörn Josewski, Stefan Dübel, Wulf Blankenfeldt, Udo Rau

**Affiliations:** 1 Structure and Function of Proteins, Helmholtz Centre for Infection Research, Inhoffenstraße 7, 38124 Braunschweig, Germany; 2 Department of Biotechnology, Institute for Biochemistry, Biotechnology and Bioinformatics, Technische Universität Braunschweig, Spielmannstraße 17, 38106 Braunschweig, Germany

## Abstract

Schizophyllan (SCH) is a high molecular weight homopolysaccharide composed of a β-(1,3)-D-glucan main chain with branching β-(1,6)-bound D-glucose residues. It forms triple helices that are highly stable towards heat and extreme pH, which provides SCH with interesting properties for industrial and medical applications. The recombinant anti-SCH antibody JoJ48C11 recognizes SCH and related β-(1,6)-branched β-(1,3)-D-glucans, but details governing its specificity are not known. Here, we fill this gap by determining crystal structures of the antigen binding fragment (Fab) of JoJ48C11 in the apo form and in complex with the unbranched β-(1,3)-D-glucose hexamer laminarihexaose 3.0 and 2.4 Å resolution, respectively. Together with docking studies, this allowed construction of a JoJ48C11/triple-helical SCH complex, leading to the identification of eight amino acid residues of JoJ48C11 (Tyr27_H_, His35_H_, Trp47_H_, Trp100_H_, Asp105_H_; Asp49_L_, Lys52_L_, Trp90_L_) that contribute to the recognition of glucose units from all three chains of the SCH triple helix. The importance of these amino acids was confirmed by mutagenesis and ELISA-based analysis. Our work provides an explanation for the specific recognition of triple-helical β-(1,6)-branched β-(1,3)-D-glucans by JoJ48C11 and provides another structure example for anti-carbohydrate antibodies.

## Introduction

Schizophyllan (SCH) is a homopolysaccharide produced by the basidiomycete *Schizophyllum commune*^1^. It is composed of a β-(1,3)-D-glucan main chain that possesses additional single β-(1,6)-bound D-glucose residues at approximately every third monomer of the backbone^2–4^. The average molecular weight ranges from 6 × 10^6^ to 1.3 × 10^7^ g moL^−1^ ^5,6^. In aqueous solution, SCH exists as a trimeric molecule with three twisted glucan chains held together by hydrogen bonds and the β-(1,6)-bound D-glucose residues pointing towards the outside^6–9^. The helical secondary structure is stable in a broad pH range and at high temperatures, starting to dissolve into single chains only at pH > 12 and above 135 °C^9,10^. The rigidity of the triplex is considered to be the prime reason for the high viscosities of aqueous SCH solutions and their pseudoplastic flow behavior, since its denaturation leads to strong reduction of viscosity^1,6,9–11^. Because of the stable viscosity at high temperatures and high salt concentrations, SCH is tested as polymer flooding agent for enhanced oil recovery^12,13^. Furthermore, SCH has several bioactive properties, similar to other fungal β-(1,3)-D-glucans^14–18^. It shows antitumoral and immunomodulating effects which are assumed to be based on the enhancement of cell-mediated immune response^19–22^.

Because of the potential of SCH in different applications, specific SCH antibodies for quantitative trace analysis or for the investigation of the role of particular conformations for glucan bioactivity are needed. Towards this, we have generated recombinant antibodies (rAbs) against SCH by antibody phage display^23^. Next to SCH, these antibodies were identified to bind also to other similar β-(1,6)-branched β-(1,3)-D-glucans like scleroglucan and cinerean^24–26^. Further specificity profiling indicated the triple helical structure with the protruding β-(1,6)-linked D-glucose as a crucial factor for binding. Denaturation of the secondary structure resulted in a sharp decrease of binding affinity and no binding was detected for β-(1,6)-D-gentiobiose and laminarihexaose representing the β-(1,6)-branches and β-(1,3)-backbone. For validation of the hypothesis that the secondary structure of SCH is indeed important for recognition, structure analysis of the antibody paratope would be required to develop a structural model that describes the interaction with SCH and related molecules.

Here, we have determined the crystal structure of the antigen binding fragment (Fab) of anti-SCH antibody JoJ48C11, both in the apo form and in complex with the oligosaccharide laminarihexaose. These structures allow modelling of an antibody/SCH complex which suggests that all three chains of the helical glucan engage in interactions with the paratope of JoJ48C11. Importantly, these interactions involve several protruding β-(1,6)-bound glucose residues of SCH, explaining the specificity of the antibody. The model was validated by introducing point mutations into JoJ48C11, corroborating its correctness.

## Results

### Structure analysis of JoJ48C11 Fab and the JoJ48C11 Fab/laminarihexaose complex

In order to obtain detailed information about the binding of SCH and other β-(1,3)-branched β-(1,6)-D-glucans by JoJ48C11, we resorted to an X-ray crystallographic structure analysis. For this, we produced recombinant Fab fragments of JoJ48C11 by adding human constant C_H_1 and C_kappa_ domains to the respective variable regions of the initial JoJ48C11 scFv fragment and crystallized the purified protein to collect diffraction data.

**Figure 1:**
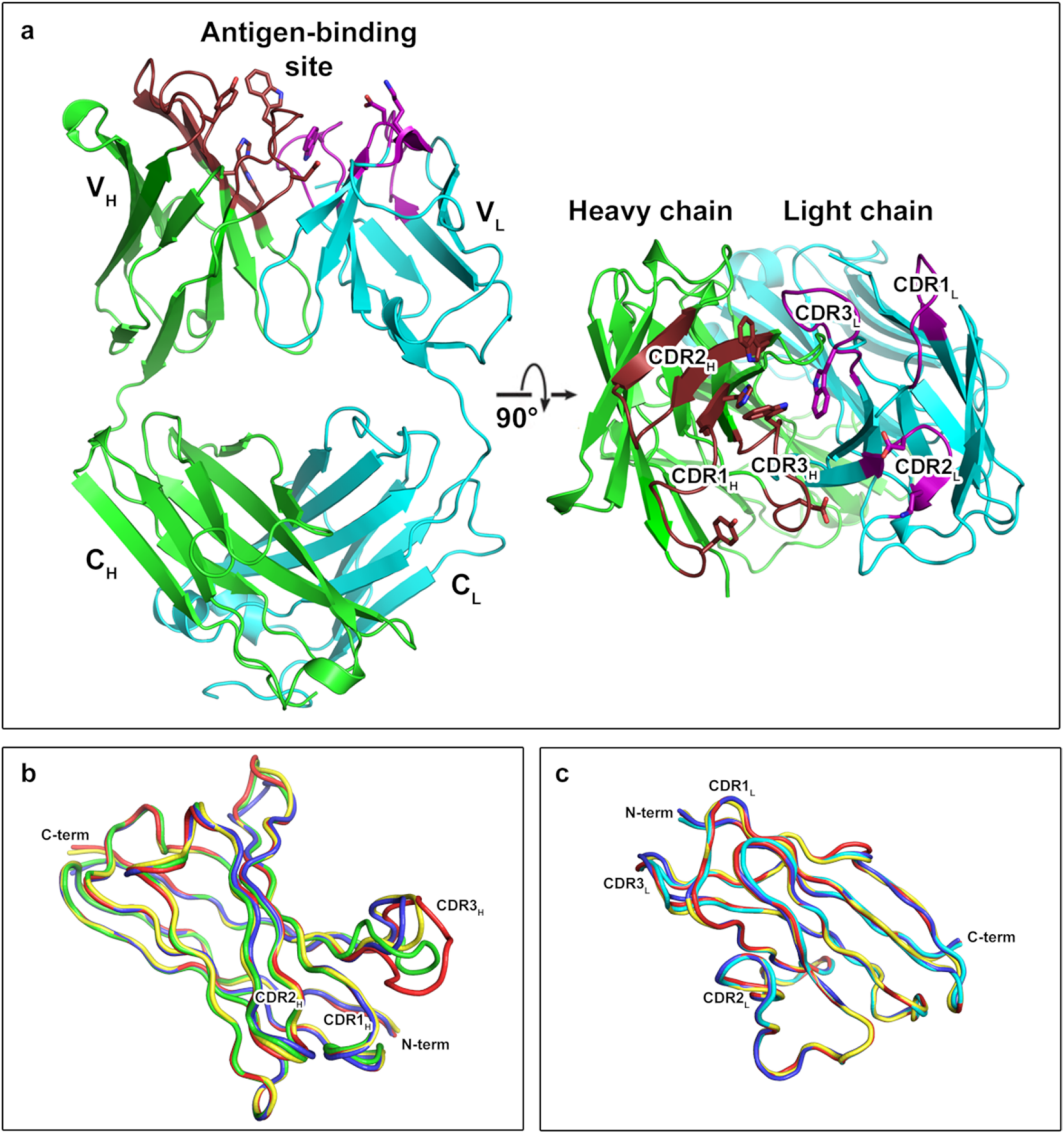
Structure of JoJ48C11 Fab. (**a**) Overall structure of JoJ48C11 Fab. The colors for each chain are green and cyan for heavy chain and light chain, respectively. CDR-region on heavy chain and light chain shown as brown and magenta colors. Sticks indicate important amino acid residues for binding to triple-helical SCH. (**b**) Superposition of variable regions of the heavy chain of JoJ48C11 and previously published Fab fragments. The colors are green for JoJ48C11, and red, blue or yellow for an antibody against SPE7 (PDB entry 1OAQ), Fab316.1 (PDB entry 5D93) or 10D10 (PDB entry 5T5N), respectively. (**c**) Superposition of the variable regions of the light chain of JoJ48C11 and previously published Fab fragments. The colors are cyan for JoJ48C11, and red, blue or yellow for an antibody against TRAAK (PDB entry 4I9W), B7-H6 (PDB entry 4ZSO) or MFE-23 (PDB entry 1QOK), respectively.

Initial phasing of the JoJ48C11 Fab fragment was performed by molecular replacement with the structure of a chimeric antibody Fab fragment against CD25 (PDB entry 1MIM) as a search model^27^. The resulting structure was refined to 3.0 Å resolution. The JoJ48C11 Fab fragment adopts the expected heterodimeric structure of a heavy chain fragment and a light chain both consisting of a constant (C) and a variable (V) immunoglobulin (Ig) domain. The Ig domains show the typical immunoglobulin fold characterized by two anti-parallel β-sheets packed against each other (Fig. 1a)^28^. Not surprisingly, backbone atoms can easily be superposed onto other Fab structures, and even several structures with almost identical C_L_, C_H_ and V_L_ domains including their CDRs can be found (Table 1). However, with respect to previously published Fab fragments, the V_H_ domain of JoJ48C11 Fab is slightly different, especially in the CDR3 region (Fig. 1b,c).

**Table 1:** Superposition of JoJ48C11 Fab on other Fab Cα-positions.

C_L_				C_H_				V_L_				V_H_			
PDB IDs	Z^a^	rmsd^b^	%id^c^	PDBIDs	Z^a^	rmsd^b^	%id^c^	PDB IDs	Z^a^	rmsd^b^	%id^c^	PDB IDs	Z^a^	rmsd^b^	%id^c^
5ILD	21.1	0.4	99	5ITB	21.1	0.5	99	4I9W	23.4	0.4	96	1OAQ	23.3	1.2	84
5VH3	21.1	0.4	99	4HH9	21.1	0.5	100	4ZSO	23.2	0.4	90	1OAU	23.2	1.1	84
4XMP	21.1	0.4	99	5U3M	21.1	0.5	99	1QOK	23.1	0.6	89	1OAR	23.1	1.2	84
4J6R	21.0	0.5	99	5IES	21.1	0.3	100	5D93	23.1	0.5	87	5D93	23.1	1.0	77
5TE7	21.0	0.5	99	5MVZ	21.1	0.5	99	2ZKH	23.1	0.5	83	2E27	23.1	0.8	78
5MVZ	21.0	0.5	99	4PUB	21.1	0.4	99	2W9D	23.1	0.6	93	5T5N	23.1	1.1	79
3QOS	21.0	0.4	99	1L7I	21.0	0.5	100	2Z91	23.0	0.5	92	3HNS	23.0	1.2	70
5DRX	21.0	0.4	99	5U3O	21.0	0.6	99	5D93	23.0	0.5	87	1SY6	23.0	1.0	89
5D6C	21.0	0.3	99	5U3N	21.0	0.5	99	4F33	23.0	0.6	90	3HNT	23.0	1.2	70
5IES	20.9	0.3	100	4IFY	20.9	0.7	100	4EBQ	23.0	0.6	83	1OAX	22.9	1.2	84

The percent identities, rmsd values, and Z-scores have been calculated with DALI^62^ using the JoJ48C11 Fab as a reference (PDB entry 6EV1).

^a^Z: Z-scores, DALI gives higher Z-scores for highly similar structures.

^b^rmsd: Root mean square deviation for superposition of Cα atoms.

^c^%id: Percent identities.

**Figure 2:**
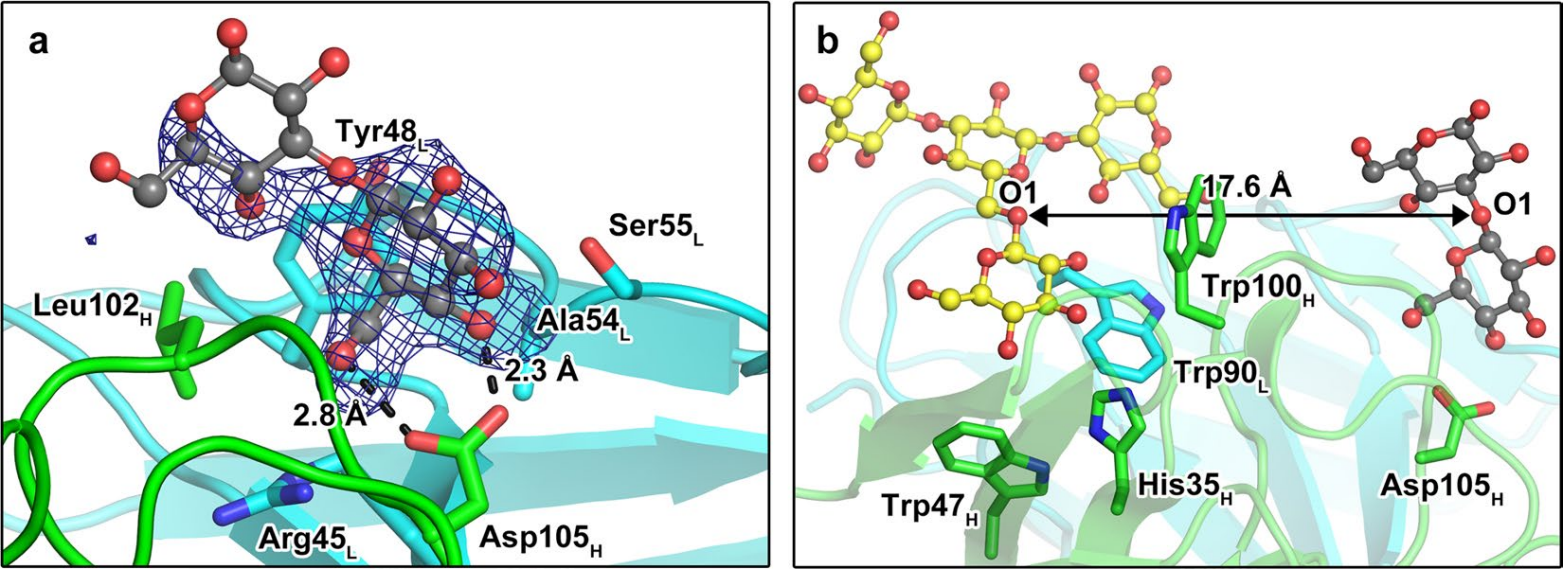
Structural models of the binding between JoJ48C11 Fab and glucose moieties. (**a**) X-ray crystal structure of JoJ48C11 Fab complex involved in laminarihexaose. Glucose residues of laminarihexaose bound to JoJ48C11 Fab are shown as ball-and-sticks (gray) with mF_o_-DF_c_ electron density map contoured at 2.5 σ. Dashed lines indicate potential hydrogen bonds. (**b**) Superposition of the laminarihexaose complex structure (gray) and the SCH RU binding model derived by docking calculations (yellow).

For understanding of the molecular determinants that contribute to the recognition of SCH by JoJ48C11, we aimed at obtaining the crystal structure of a complex with SCH but failed despite extensive attempts. Therefore, we resorted to structure determination of a complex with the less complex oligosaccharide laminarihexaose, which is a β-(1,3)-linked unbranched glucose hexamer. Electron density for two glucose moieties was observed when crystals were soaked with high concentration of the ligand (50 mM laminarihexaose) for one week. Interestingly, laminarihexaose does not show binding to JoJ48C11 in an *in vitro* affinity assay^23^, indicating that this is a good example for the sensitivity of protein crystallography to detect lower affinity^29^. The JoJ48C11 Fab/laminarihexaose structure was refined to 2.4 Å resolution. In the complex, Asp105_H_ of the heavy chain CDR3 region binds the O4- and O6-atoms of the non-reducing end of laminarihexaose in a bidentate fashion (Fig. 2a and Supplementary Fig. S1). To confirm the observed interaction of Asp105_H_, we generated an alanine mutant and produced it as scFv-Fc. Binding of JoJ48C11-Asp105Ala_H_ scFv-Fc to SCH was completely abrogated in a titration ELISA, which corroborates the importance of Asp105_H_ and the significance of the JoJ48C11 Fab/laminarihexaose complex crystal structure.

The apo structure of JoJ48C11 Fab and the complex with laminarihexaose were used as template for modeling of JoJ48C11 Fab in complex with the triple helical SCH as described below.

### Modelling the interaction of JoJ48C11 with triple helical SCH

To further refine insight into the JoJ48C11 Fab/SCH interaction, we used SwissDock to generate models of a complex with the repeating unit (RU) of SCH and the apo structure of JoJ48C11 Fab. 256 models in 44 clusters were generated and then inspected to select the most likely models for further analysis. Two similar docking poses positioning the RU inside the typical antigen binding area of antibodies were identified. They proposed that Trp33_H_, His35_H_, Trp47_H_, Tyr50_H_, Glu59_H_, Trp100_H_, Trp90_L_ and Asn93_L_ were interacting residues. Subsequent mutagenesis studies proved His35_H_, Trp47_H_, Trp100_H_ and Trp90_L_ to be necessary for binding SCH, since their respective alanine mutants showed significant decrease of binding strength with EC50 values of 1.1 nM to 10.9 nM, which are higher than 0.6 nM (3 times the EC50 value of wild type JoJ48C11 scFv-Fc). It is important to note that the main interaction sites predicted by docking are different from the position identified in the crystal structure with laminarihexaose, suggesting that JoJ48C11 may contain two separated recognition motives for β-(1-6)-linked glucose moieties (Fig. 2b).

**Figure 3:**
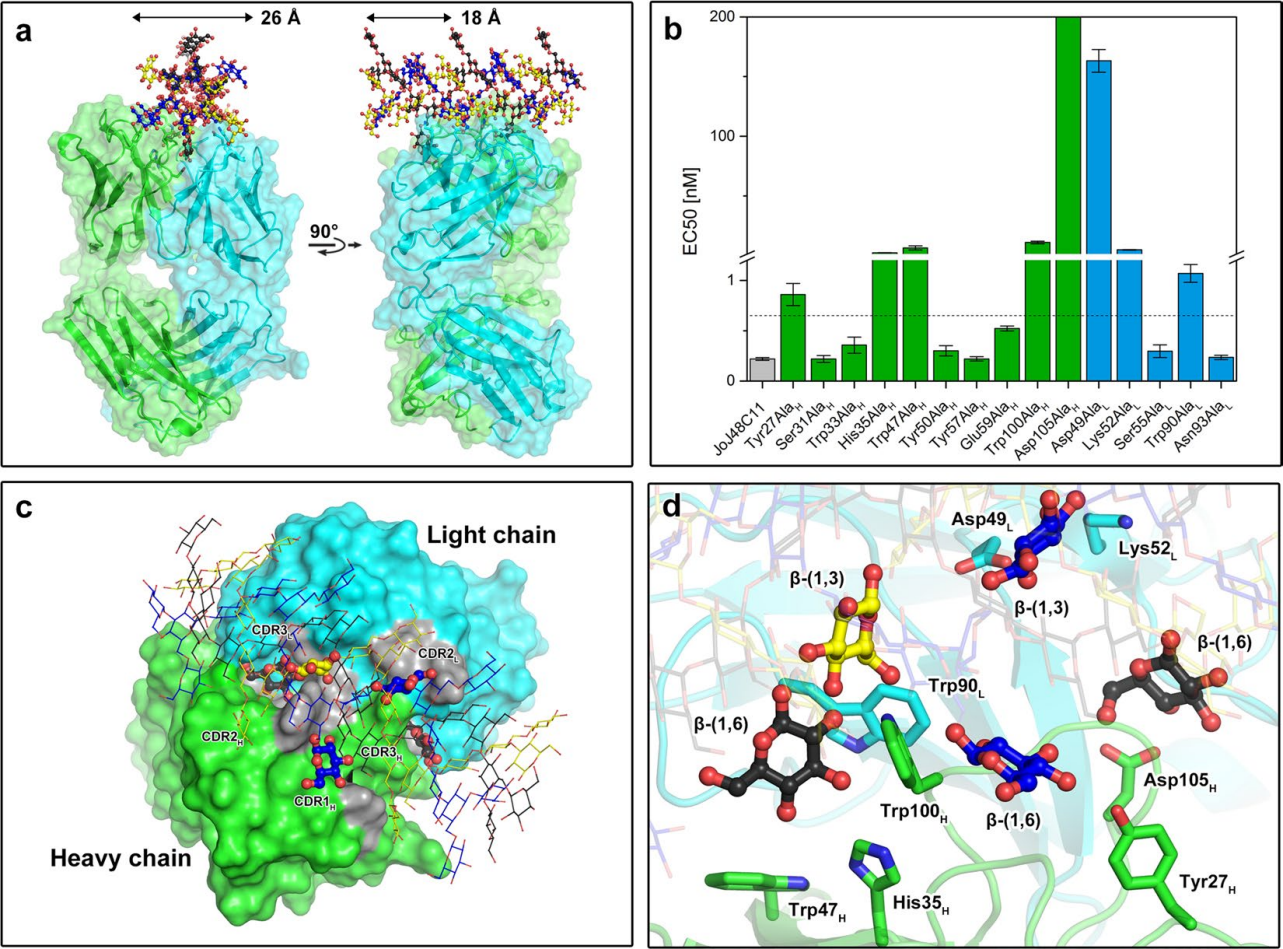
Model of the complex between JoJ48C11 Fab and triple-helical SCH. (**a**) Overall view. The chains of SCH are colored black, blue and yellow, respectively for chain 1, 2 and 3. (**b**) Comparison of the calculated EC50 values from the Binding assay with SCH and point mutants of JoJ48C11 in the scFv-Fc format. The dashed line at 0.6 nM marks the EC50 value considered to be the threshold for indicating a significant decrease in binding strength. (**c**) Surface model of JoJ48C11 Fab in complex with triple-helical SCH. The CDR-regions of the antigen binding site are shown in gray and thick ball-and-sticks indicate important glucose moieties interacting with JoJ48C11 Fab. (**d**) Close-up view of the triple-helical SCH binding site of JoJ48C11 Fab.

With the information from the crystal complex and the docking poses with the RU of SCH, we aimed at generating a model of JoJ48C11 in complex with triple helical SCH. Interestingly, the binding sites for the terminal glucose moiety of laminarihexaose observed in the crystal structure and the second binding site identified by docking calculation are approx. 18 Å apart, which corresponds very well to the helix pitch distance of the SCH chains in the triple-helical form (Figs 2b and 3a)^30^. We therefore created a model of JoJ48C11 Fab in complex with triple-helical SCH by superimposing a SCH triple helix structure from the literature onto the β-(1,6)-linked glucose unit of the docked SCH RU (Supplementary Fig. S2)^31^. To corroborate this model, we examined additional residues that are located close to the predicted binding sites of the triple-helical SCH, namely Tyr27_H_, Ser31_H_, Tyr57_H_, Asp49_L_, Lys52_L_ and Ser55_L_, by generating respective alanine exchange mutants in the scFv-Fc format. This revealed Tyr27_H_, Asp49_L_ and Lys52_L_ to be significantly involved in binding of SCH, validating our structural model of the JoJ48C11 Fab/triple-helical SCH interaction (Fig. 3b and Table 2).

**Table 2:** EC50 values from the Titration ELISA of JoJ48C11 scFv-Fc and its point mutants.

scFv-Fc	EC50 [nM]	scFv-Fc	EC50 [nM]
JoJ48C11	0.22 ± 0.01	Glu59Ala_H_	0.52 ± 0.03
Trp27Ala_H_	0.86 ± 0.11	Trp100Ala_H_	10.94 ± 1.15
Ser31Ala_H_	0.22 ± 0.04	Asp105Ala_H_	>>1000
Trp33Ala_H_	0.36 ± 0.08	Asp49Ala_L_	163.2 ± 9.5
His35Ala_H_	2.28 ± 0.17	Lys52Ala_L_	4.78 ± 0.26
Trp47Ala_H_	6.40 ± 1.17	Ser55Ala_L_	0.30 ± 0.06
Tyr50Ala_H_	0.30 ± 0.05	Trp90Ala_L_	1.07 ± 0.09
Tyr57Ala_H_	0.22 ± 0.02	Asn93Ala_L_	0.23 ± 0.02

The model suggests that five glucose units are involved in binding to JoJ48C11: two β-(1,6)-linked moieties of SCH chain 1 fit into the two binding cavities found in the crystal structure with laminarihexaose and in the docking studies with the RU of SCH, one β-(1,6)-linked and one β-(1,3)-linked glucose group of SCH chain 2 interact with V_H_ and V_L_ in the area between the two binding cavities, and one β-(1-3)-linked moiety of SCH chain 3 interacts with V_L_ near the docking site of the RU of SCH. The distribution of torsion angles values at the β-(1,3)-glycosidic bonds of triple-helical SCH in the modeled complex with JoJ48C11 after energy minimization is very similar to values observed in crystal structures, indicating the high geometrical quality of the model (Supplementary Fig. S3). According to our binding studies, the antibody itself contributes eight amino acid residues (Tyr27_H_, His35_H_, Trp47_H_, Trp100_H_, Asp105_H_; Asp49_L_, Lys52_L_, Trp90_L_) to this interaction, acting mainly through hydrogen bonds or π-effects (Fig. 3c,d). Taken together, this provides an explanation for JoJ48C11’s specificity towards SCH triple helices.

## Discussion

Basic characteristics for the paratope structure of carbohydrate binding antibodies were early established by studies of anti-dextran binding antibodies. They predicted that the respective antibodies consist of a cavity-shaped paratope for accommodation of terminal saccharide residues or of a groove structure for up to seven internal saccharide residues of a linear polysaccharide chain^32,33^. To date, the structures of a few anti-carbohydrate antibodies have been determined by X-Ray crystallography. They indeed show the predicted cavity or groove structures of their paratope and some even consist of a combined structure of both types^34^. To name one interesting example, the monoclonal antibody F22-4 that is directed against the O-Antigen (lipopolysaccharide) from serotype 2a *Shigella flexneri* consist of a groove-shaped paratope with two small cavities integrated. Co-crystallization with a synthetic fragment (decasaccharide) of the O-antigen clearly showed the binding-orientation of the helically bowed fragment along the groove with two branches of single glucose residues fitting into the small cavities^35^. For JoJ48C11 we stated that the combination of the triple helical structure with the protruding β-(1,6)-linked D-glucose is a crucial factor for binding^23^. Therefore, we expected a groove-shaped paratope for interchain recognition of SCH with at least one cavity-shaped binding site to accommodate a β-(1,6)-linked D-glucose.

Here, we discovered via protein crystallography and *in silico* docking that the paratope structure of JoJ48C11 consists of two cavity-shaped binding sites that are connected by a shallow groove. These cavities can accommodate single glucose residues and are approx. 18 Å apart, a distance that corresponds strikingly well to the helical pitch in structural models of triple-helical SCH^30^. This enabled us to generate a model of the JoJ48C11 Fab/SCH complex, which is defined by the protruding β-(1,6)-linked D-glucose residues positioned in the two cavities. Experimental validation by mutagenesis indicated that eight amino acids of JoJ48C11 predicted to be involved in binding of SCH, namely Tyr27_H_, His35_H_, Trp47_H_, Trp100_H_, Asp105_H_, Asp49_L_, Lys52_L_ and Trp90_L_, are indeed required, confirming the correctness of the model.

The complete abrogation of binding by mutation of Asp105_H_ reveals the critical function of this residue. As part of one of the two binding cavities, it was identified to engage in two hydrogen bonds with the glucose unit at the non-reducing end of laminarihexaose in the complex crystal structure. Such bidentate interactions between acidic residues and saccharide moieties are an important motif in protein/carbohydrate complexes^36^ and the model with SCH suggests that Asp105_H_ contributes a similar interaction to a β-(1,6)-linked moiety from SCH chain 1.

*In silico* docking suggested that His35_H_, Trp47_H_, Trp100_H_ and Trp90_L_ establish walls around a second cavity for the binding of a second β-(1,6)-linked glucose moiety of SCH chain 1. Histidine and tryptophan are often found in carbohydrate binding sites, where they seem to interact through aromatic contacts^36^. Such interactions are known to determine the specificity of carbohydrate binding proteins, but their contribution to the stability of such complexes is rather modest^37,38^, which is in line with the observation that mutation of these residues led to a smaller affinity decrease than in the case of the Asp105Ala_H_ substitution. However, the function of Trp47_H_ and Trp100_H_ may extend beyond establishing aromatic contacts. As a conserved residue in frame work 2 of the VH domain, Trp47_H_ seems to possess an additional structural role: Herold *et al*. observed that Trp47_H_ contributes to the stability of the V_H_ domain as well as to the V_H_/V_L_ association in the antibodies MAK33 and 2A2 rheumatoid factor^39^. Trp100_H_, on the other hand, seems to involve the an additional hydrogen bond between the indole group and a β-(1,3)-glucose moiety of SCH chain 3. A similar interaction has also been observed by Cygler *et al*. for an antibody against polysaccharide O-antigen of pathogenic *Salmonella* of serogroup B^40^. Interestingly, in order to avoid clashes with SCH, Trp100_H_ has to adopt a different sidechain rotamer than is observed the apo structure. The required conformation is found in two out of four copies of the laminarihexaose complex, however, which may be indicative of crosstalk between the two glucose binding sites (Supplementary Fig. S4).

The potential involvement of Tyr27_H_, Asp49_L_ and Lys52_L_ in SCH recognition was only discovered from the JoJ48C11/triple-helical SCH complex model. Tyr27_L_ seems to interact with a β-(1,6)-bound glucose of SCH chain 2, whereas Asp49_L_ and Lys52_L_ engage in hydrogen bonds with one β-(1,3)-bound glucose unit of the same SCH chain. Of these residues, Asp49_L_ is especially important as can be concluded from the large decrease in binding affinity observed after its mutation to alanine (Fig. 3b).

In summary, we have determined the structure of the anti-SCH antibody JoJ48C11 and obtained further insight into SCH binding by combining crystal structure analysis of a complex with a molecule that does not show binding in ELISA experiments, *in silico* docking of a SCH RU and modeling of the JoJ48C11 Fab/triple-helical SCH complex by hand. The model is consistent with experimental data and validates our previous hypothesis about the specificity of JoJ48C11 towards SCH and other similar β-(1,6)-branched β-(1,3)-D-glucans, namely that the β-(1,6)-bound D-glucose residues in conjunction with the triple-helical structure constitute the binding epitope for the antibody JoJ48C11^23^. Our work provides first insight into the structural principles that govern the binding of triple-helical SCH to a SCH-specific antibody and provides another example of an anti-carbohydrate antibody structure in complex with its antigen.

## Materials and Methods

### Preparation of JoJ48C11 as Fab for crystallography

JoJ48C11 was produced as a Fab in HEK239–6E cells (National Research Council, Biotechnological Research Institute). Towards this, the DNA encoding V_H_ and V_L_ of JoJ48C11 scFv were cloned into the respective vectors pCSE2.5-Fab-k-His-XP and pCSE2.5-Fab-h-His-XP^23^. A mixture containing 12.5 µg of both vectors was used for PEI-mediated transfection of 25 mL culture (about 1.5 × 10^6^ cells mL^−1^). Transfection and production were carried out as described previously^23,41^. After production, the Fab was isolated by His-tag affinity purification. 1 mL of Chelating Sepharose Fast Flow (GE Healthcare) complexed with nickel was incubated with the cell free supernatant supplemented with 0.5 M NaCl. The sepharose was washed in a gravity flow column with 25 volumes of His-tag binding buffer (0.5 M NaCl, 20 mM Na_2_HPO_4_, 20 mM NaH_2_PO_4_, 10 mM imidazole, pH 7.4) and the Fab was eluted in 2 mL 0.1 M EDTA in PBS (8.0 g L^−1^ NaCl, 0.2 g L^−1^ KCl, 1.44 g L^−1^ Na_2_HPO_3_*4 H_2_O, 0.24 g L^−1^ KH_2_PO_4_, pH 7.4). Possible agglomerates were removed by subsequent size exclusion chromatography. For this, the eluate of the His-tag affinity purification was loaded onto a Superdex 75 HiLoad 16/60 column (GE Healthcare) running at 0.75 mL min^−1^ in crystallization buffer (20 mM Tris-HCl, 150 mM NaCl, pH 8.0). The resulting monodisperse Fab was concentrated to 7.2 mg mL^−1^ with Amicon Ultra – 0.5 mL Centrifugal Filters 30 K (Merck Millipore) and directly used for crystallization.

### Crystallization and data collection

Initial crystallization experiments were carried out with a HoneyBee crystallization robot (Digilab) and 96-well sitting-drop Intelli-plates (Art Robbins Instruments) at 200 nL volume of protein and an equal volume of reservoir solution. Clusters of needle-shaped multicrystals growing from a single nucleation center were obtained at 20 °C with a precipitant containing 0.1 M Tris pH 8.5, 0.2 M lithium sulfate and 30% w/v PEG 4000. Crystals were washed in cryoprotectant (0.1 M Tris pH 8.5, 0.2 M lithium sulfate, 30% w/v PEG 4000 and 20% w/v glycerol) before flash-cooling in liquid nitrogen. A single needle was isolated and a 3 Å data set was collected from two wedges at 100 K on beamline P11 of the PETRAIII-synchrotron at DESY (Hamburg, Germany), using a PILATUS 6M-F detector to merge 1800 and 400 0.1°-frames, respectively. The crystal belonged to the monoclinic space group P2_1_, with cell parameters of a = 94.03 Å, b = 112.86 Å, c = 140.82 Å and β = 98.62°. The asymmetric unit contained 6 copies of Fab fragment with a calculated Matthews parameter (V_M_) of 2.41 Å^3^/Da, corresponding to a solvent content of 48.9%.

For optimization of the crystallization, macroseeding was carried out with drops consisting of 500 nL protein solution, 600 nL precipitant (0.1 M HEPES pH 7.5, 0.1 M sodium acetate, 0.2 M lithium sulfate, 25% w/v PEG 4000) and 100 nL macroseed crystal from 0.1 M Tris pH 8.5, 0.2 M lithium sulfate and 30% w/v PEG 4000, set up with an OryxNano crystallization robot (Douglas Instruments Ltd). Crystals were observed after 2 months and were soaked by adding 50 mM laminarihexaose to the drop for 1 week and subsequent flash freezing in liquid nitrogen without additional cryoprotectant. Diffraction data were collected from 3600 0.05°-oscillation images to a maximum resolution of 2.4 Å. The complex crystal belonged to space group P2_1_ with cell parameters of a = 81.73 Å, b = 131.50 Å, c = 91.03 Å and β = 91.51°. The asymmetric unit contained 4 copies of the Fab fragment, with a calculated Matthews parameter (V_M_) of 2.39 Å^3^/Da (solvent content 48.5%). Diffraction data were processed and scaled using autoPROC with XDS, pointless and aimless^42–46^. Statistics for the data collection are described in Table 3.

**Table 3:** Data collection and refinement statistics.

	JoJ48C11 Fab	JoJ48C11 Fab + laminarihexaose
**Data collection**		
X-ray sources^a^	P11, DESY	P11, DESY
Space group	P2_1_	P2_1_
**Cell dimensions**		
a, b, c (Å)	94.08 112.83 140.82	81.73 131.50 91.03
α, β, γ (°)	90.00, 98.61, 90.00	90.00, 91.50, 90.00
No. of subunits/ASU^b^	6	4
Wavelength (Å)	1.03320	1.03320
Resolution (Å)	3.04 (3.096–3.043)^c^	2.40 (2.445–2.403)^c^
Total number of observation	188636 (9678)	516202 (26154)
Total number unique	55718 (2768)	74793 (3694)
*R*_sym_ (%)^d^	13.0 (64.7)	9.6 (77.9)
*R*_meas_ or *R*_r.i.m._ (%)^e^	15.5 (76.4)	10.3 (84.0)
*R*_pim_ (%)^f^	8.3 (40.4)	3.9 (31.2)
Mean(I)/sd(I)	8.9 (2.2)	12.9 (2.2)
Completeness (%)	99.4 (99.3)	100.0 (99.6)
Multiplicity	3.4 (3.5)	6.9 (7.1)
**Refinement**		
Resolution range (Å)	56.416–3.043	55.787–2.403
Reflections used	55674	74686
*R*_*work*_/*R*_*free*_ (%)^g^	18.56/23.79	19.35/23.29
**Number of atoms**		
Protein	19269	13102
Ligand		35
Water	9	529
**Average** ***B*** **factors (Å** ^**2**^ **)**		
Protein	66.58	57.18
Ligand		97.28
Water	35.30	49.31
**RMS deviations**		
Bond length (Å)	0.004	0.004
Bond angles (°)	1.003	0.756
Ramachandran outliers (%)	0.08	0.23
PDB ID	6EV1	6EV2

^a^DESY: German Electron Synchrotron, Germany.

^b^ASU; Asymmetric Unit.

^c^Values in parentheses are for reflections in the highest resolution bin.

^d^*R*_*sym*_ = Σ_*h*_Σ_*i*_|I_*(h*,*i)*_ − <I_*(h)*_> |/Σ_*h*_Σ_*i*_ *I*_*(h*,*i)*_, where *I*_*(h*,*i) i*_s the intensity of the *i*^th^ measurement of reflection *h* and <*I*_*(h)*_> is the corresponding average value for all *i* measurements.

^e^*R*_*meas*_ = *R*_r.i.m._ (redundancy-*i*ndependent merging R-factor) = Σ_*h*_[N/(N − 1)]^1/2^Σ_*i*_(|*I*_*i(h)*_ − <*I*_*(h)*_> |)/Σ_*h*_Σ_*i*_*I*_*i(h)*_

^f^*R*_p.i.m._ (precision-indicating merging R-factor) = Σ_*h*_[1/(N − 1)]^1/2^Σ_i_(|I_*i(h)*_ − <I_*(h)*_>|)/Σ_*h*_Σ_*i*_*I*_*i(h)*_.

^g^*R*_*work*_ = Σ||*F*_*o*_| − |*F*_*c*_||/Σ|F_*o*_|, where *R*_*free*_ is calculated for the 5% test set of reflections.

### Structure determination and refinement

The structure of the anti-SCH JoJ48C11 Fab was solved by the automated molecular replacement pipeline BALBES (https://www.ccp4.ac.uk/ccp4online/), using the structure of a Fab fragment of a chimeric antibody against CD25 (PDB entry 1MIM) as a search model^27^. The complex structure with laminarihexaose was solved by molecular replacement with PHASER as included in the PHENIX software suite using the apo structure of JoJ48C11 Fab as a search model^47,48^. An initial atomic model was manually improved using COOT and refined with PHENIX and REFMAC by standard protocols^49,50^. Refinement statistics for the JoJ48C11 Fab fragment structures are summarized in Table 3. Coordinates and structure factors have been deposited in the Protein Data Bank (PDB) with accession codes 6EV1 (JoJ48C11 Fab) and 6EV2 (JoJ48C11 Fab:laminarihexaose complex)^51^. Figures were prepared using PYMOL^52^.

### Molecular modeling

Molecular docking calculations were performed using the SwissDock web service (http://swissdock.vital-it.ch/) with default parameters and settings^53,54^. The variable region of the apo structure of JoJ48C11 Fab (residue 1 to 116 of chain A (V_H_) and 1 to 106 of chain B (V_L_)) was selected as the target. Water molecules were removed from the structure. For the ligand, a three-dimensional model of the SCH RU was obtained from the PubChem Compound Database (CID:24777) and was converted into MOL2 file format using YASARA View^55,56^. Results were visualized with the UCSF Chimera package and unique docking poses were selected by visual inspection^57^.

To derive a model for the interaction of JoJ48C11 with triple-helical SCH, the selected docking pose of the SCH RU was superposed onto chain A and B of the laminarihexaose complex. Residues Leu99 to Asp105 of chain A and Lys44 to Val57 as well as Gln89 to Asn93 of chain B were replaced with the corresponding residues from chain A and B of the apo structure due to the fact that Trp100 of chain A and Asp49 and Trp90 of chain B adopted different sidechain rotamers than in the apo structure that had been used for the docking calculations with SCH RU. The model of triple-helical SCH was generated using a structure of the triple-helical curdlan as a template (http://polysac3db.cermav.cnrs.fr/polysacdb/curdlan-II/curdlan2_3chains_expanded.pdb in PolySac3DB), which was modified into triple-helical SCH by manually adding β-(1,6)-bound glucose moieties homogenously at every third glucose moiety of each chain^58,59^. Subsequently, triple-helical SCH was fitted onto the JoJ48C11 Fab structure by visual superposition of two β-(1,6)-bound glucose with the two glucose moieties from the complex structure with laminarihexaose and the docking model with SCH RU. Subsequently, the two β-(1,6)-bound glucose of triple-helical SCH used for the superposition were replaced with the two glucose moieties from the laminarihexaose complex and the SCH RU docking pose. Finally, energy minimization was performed with the YASARA Energy Minimization Server (http://www.yasara.org/minimizationserver.htm)^60^. The complete docking and modeling procedure is summarized in Supplementary Fig. S1.

### Generation and preparation of JoJ48C11 mutants as scFv-Fc

The mutants of JoJ48C11 variable chains for the binding studies were derived by quick change PCR for site directed mutagenesis^61^. PCR experiments were conducted with Phusion® High Fidelity DNA Polymerase (Thermo Fisher Scientific). Primer pairs were designed for each mutation site with the mismatch alanine codon framed by at least six bases in the 5′-direction and thirteen bases in the 3′-direction. The mutated scFv DNA sequence was cloned into pCSE2.6-mIgG2c-Fc-XP vector and used for transfection of HEK cells (EXPI293-F™, Thermo Fisher Scientific)^41^. The cells were cultivated as described above. For transfection, 5 µg of the vector DNA was added to 250 µL culture medium and mixed with 250 µL medium containing 25 µg polyethylenimine. 5 mL of cells (ca. 1.5 × 10^6^ cells mL^−1^) were transferred into 40 mL screw cap tubes (Sarstedt), the DNA-PEI mixture was added and the culture was incubated at 37 °C, 190 rpm as well as 5% CO_2_ in the atmosphere (HERAcell™, Thermo Fisher Scientific and Celltron, Infors). 48 h after transfection the culture was fed with 5 mL HEK TF medium (Xell AG) supplemented by 8 mmol L^−1^ L-glutamine and 1 mL of HEK FS medium (Xell AG). The scFv-Fc mutants were isolated by protein A affinity purification. 9.5 ml of cell free supernatant was incubated with 500 µL of protein A purification resin (MabSelect™ SuRe™, GE Healthcare). The loaded resin was washed with 20 volumes PBS and the scFv-Fc was eluted in 2 mL of 100 mM citrate puffer (pH 3). For pH neutralization, 220 µL of 1 M Tris was added immediately after elution. Afterwards the buffer was exchanged for PBS by centrifugation with Zeba™ desalt spin columns (Thermo Fisher Scientific). The concentrations of the antibody preparations were calculated by their absorbance at 280 nm (NanoDrop 2000, Thermo Fisher Scientific).

### Titration ELISA

Titration ELISA (enzyme-linked immunosorbent assay) was performed on Carbo-BIND™ multiwell plates (Corning) loaded with proteinase K-treated SCH (SCH-PK) as described before^23^. Briefly, serial dilutions of the scFv-Fc antibodies ranging from 0.03 pM to 950 nM were prepared in blocking solution (2% w/v milk powder and 0.05% w/v Tween® 20 in PBS) and added to the blocked multiwell plates (100 µL per well). After 1 hour incubation, the plates were washed 3 times with PBST (0.05% w/v Tween® 20 in PBS). For the titration of the point mutants in the scFv-Fc format, the plates were incubated with peroxidase-conjugated goat anti-murine Fc antibody (A0168, Sigma-Aldrich, 1:40,000 in blocking solution, 100 µL per well). For Detection of JoJ48C11 in the Fab format, the plates were first incubated with a mouse anti-His-tag antibody (DIA-900, Dianova, 100 µL per well, 40 ng ml^−1^) before incubation with the peroxidase-conjugated antibody. The ELISA was developed by adding 100 µL TMB solution (20 volumes 30 mM potassium citrate, 0.5 M citric acid, pH 4.1 and 1 volume 10 mM 3,3′,5,5′-tetramethylbenzidine, 10% v/v acetone, 90% v/v ethanol, 0.3% v/v H_2_O_2_)) per well. The color reaction was stopped after 10 minutes by addition of 100 µL of 0.5 M H_2_SO_4_ per well. The resulting absorption was measured at 450 nm (reference wavelength 620 nm). Each mutant was investigated in three independent experiments.

The datasets generated during and/or analyzed during the current study are available from the corresponding author on reasonable request.

## Electronic supplementary material

Supplementary pictures
