# Supplementary material for: Structural insights into antigen recognition of an anti-β-(1,6)-β-(1,3)-D-glucan antibody
Source: Sci Rep. 2018 Sep 12;8:13652. doi: 10.1038/s41598-018-31961-x (PMC6135813; doi:10.1038/s41598-018-31961-x)
Supplement: Supplementary file 1 — Supplementary pictures [file 41598_2018_31961_MOESM1_ESM.pdf]

Structural insights into antigen recognition of an anti-β-(1,6)-β-(1,3)-D-glucan antibody.

Authors:

Kwang Hoon Sung[1*], kwanghoon.sung@helmholtz-hzi.de, ORCiD: 0000-0002-5186-3418

Jörn Josewski[2*], j.josewski@tu-bs.de, ORCiD: 0000-0003-2469-1574

Stefan Dübel[2], s.duebel@tu-bs.de, ORCiD: 0000-0001-8811-7390

Wulf Blankenfeldt[1,2], wulf.blankenfeldt@helmholtz-hzi.de, ORCiD: 0000-0001-9886-9668

Udo Rau[2] (corresponding author), U.Rau@tu-bs.de, ORCiD: 0000-0002-8728-1495

[1]Structure and Function of Proteins, Helmholtz Centre for Infection Research, Inhoffenstraße 7, 38124 Braunschweig, Germany

[2]Department of Biotechnology, Institute for Biochemistry, Biotechnology and Bioinformatics, Technische Universität Braunschweig, Spielmannstraße 17, 38106 Braunschweig, Germany

* The two authors contributed equally to this work.

Supplementary Figures

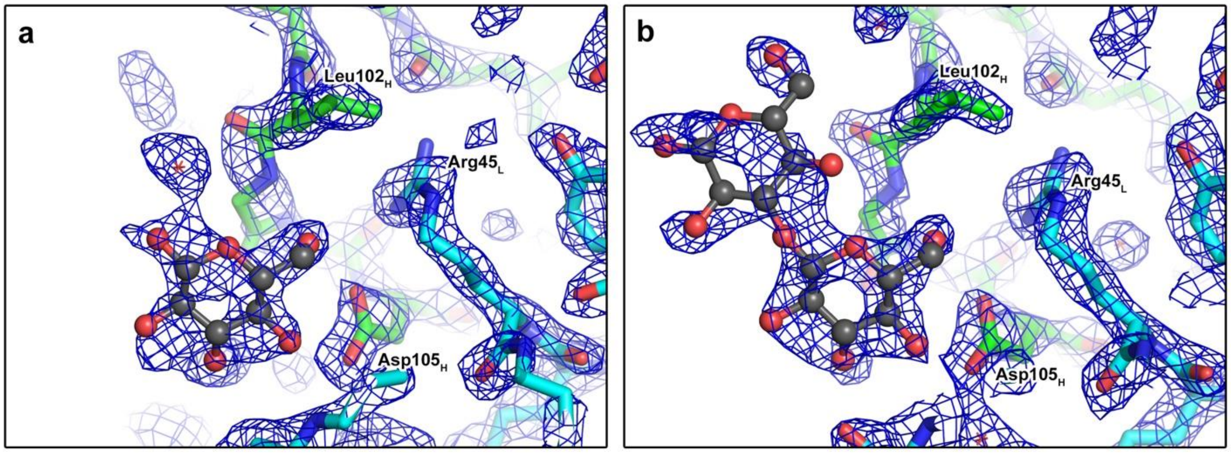

Supplementary Figure S1. A Feature Enhanced Map computed by PHENIX-FEM contoured at 2.0σ.[1] a) Chain A and B in the laminarihexaose complex. b) Chain C and D in the laminarihexaose complex. Glucose residues of laminarihexaose are shown as ball-and-sticks (gray). The heavy chain and light chains are colored in green and cyan, respectively.

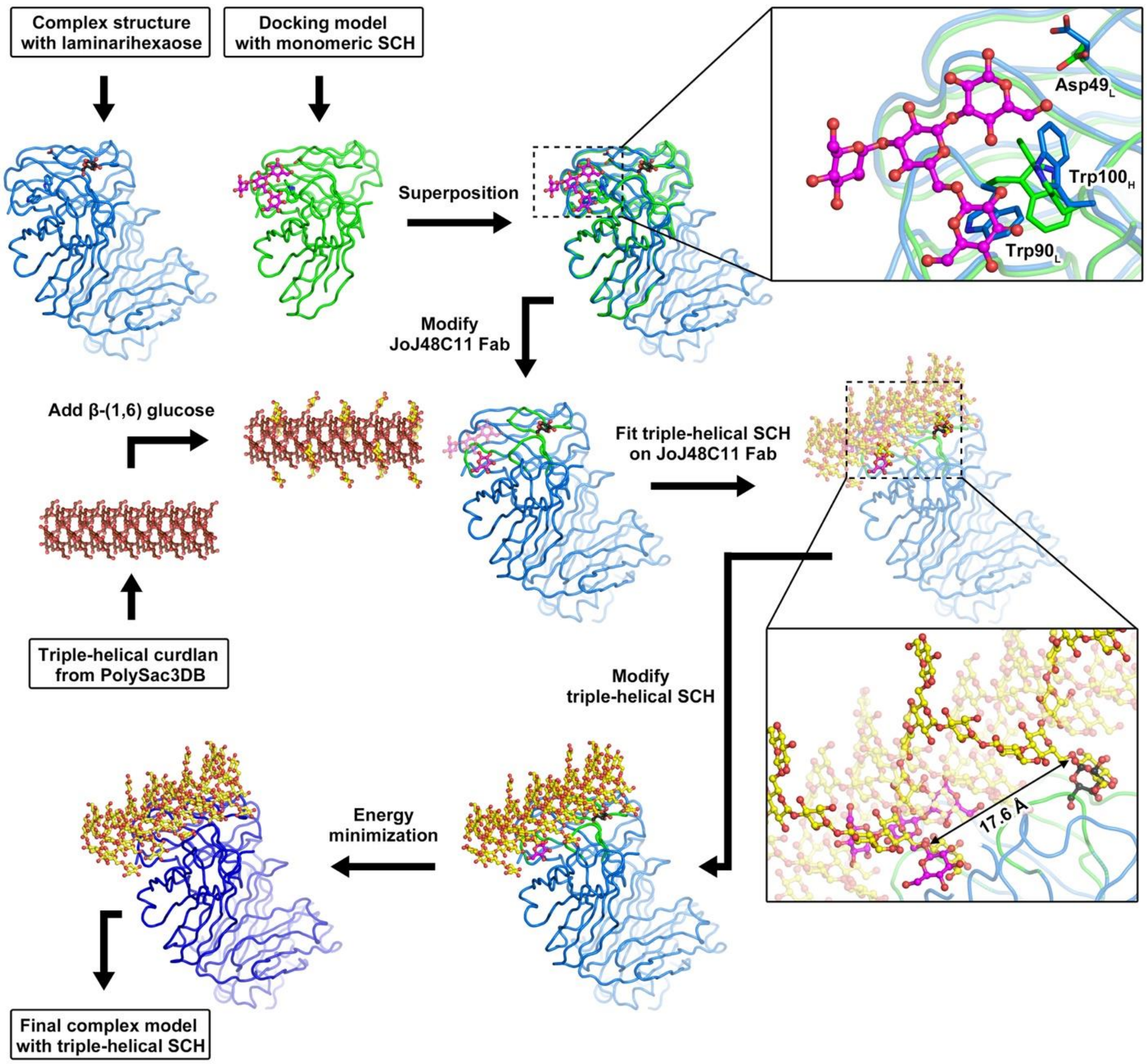

Supplementary Figure S2. Procedure for the modeling of a complex between JoJ48C11 Fab and triple-helical SCH.

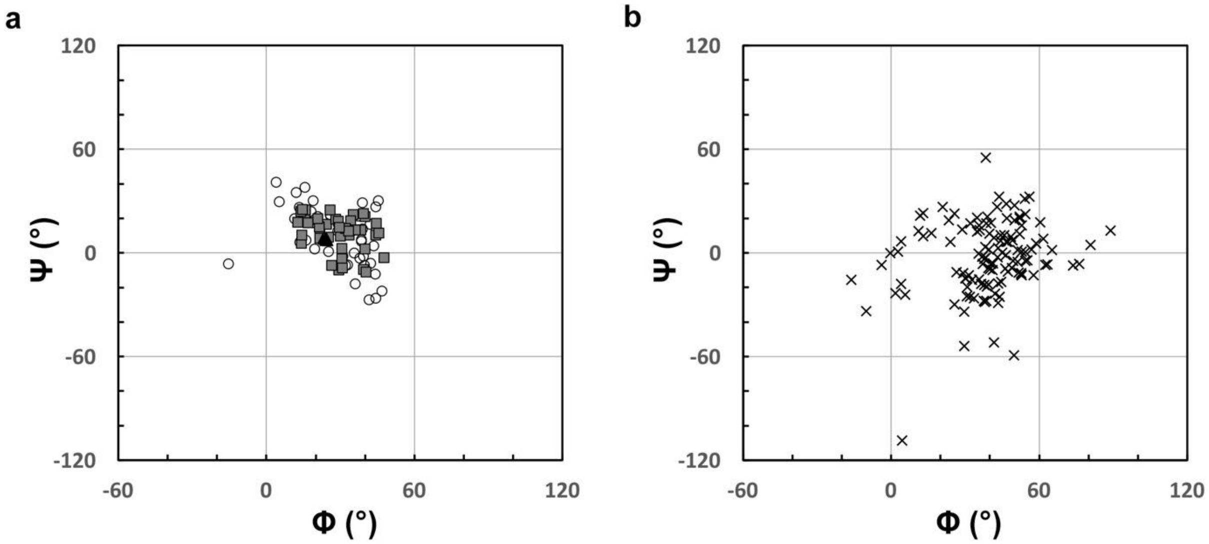

Supplementary Figure S3. Dihedral angles of β-(1,3)-glycosidic bonds. a) The *φ* (H1'-C1'-O-C3) and *ψ* (C1'-O-C3-H3) angles of the X-ray crystal structure of triple helical curdlan (▲), the energy-minimized triple helical SCH model before (□) and after docking to JoJ48C11 Fab (○). The torsion angles were measured using COOT. b) The dihedral angles of β-(1,3)-glycosidic bonds in the Protein Data Bank as summarized by GLYCOSCIENCES.de.[2]

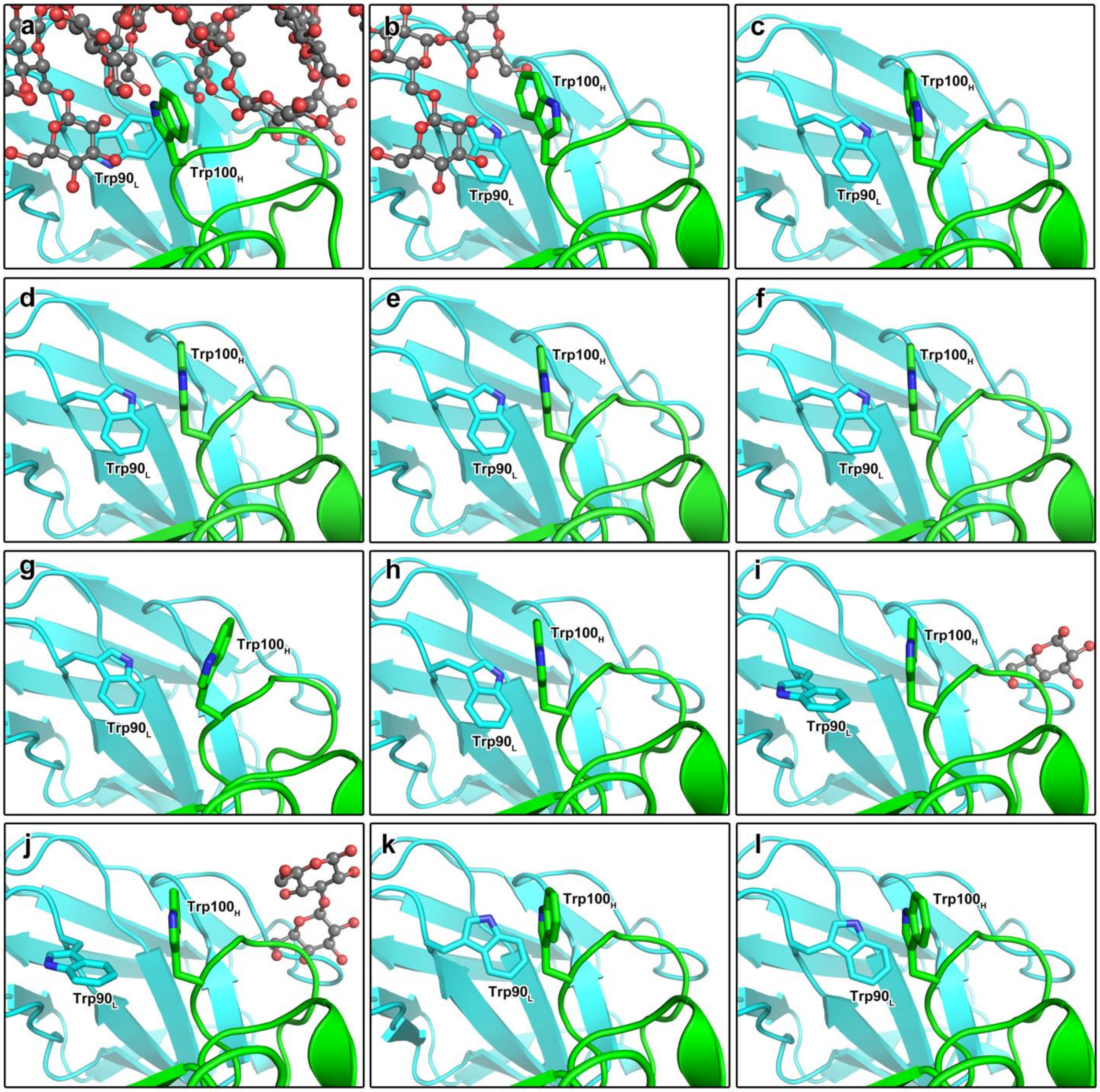

Supplementary Figure S4. Different sidechain rotamers of Trp100$_H$. a) Triple-helical SCH binding model after energy minimization. b) Monomeric SCH binding model from in silico docking. c-h) Conformations observed in the apo structure. i-l) Conformations observed in the laminarihexaose complex. Heavy chain and light chain are shown in green and cyan, respectively. Glucose residues of SCH and laminarihexaose are colored gray.

References

1. Afanine, P. V. *et al.* FEM: feature enhanced map. *Acta Crystallogr. D Biol. Crystallogr.* **71**, 646-666 (2015).
2. Lütteke, T. *et al.* GLYCOSCIENCES.de: an Internet portal to support glycomics and glycobiology research. *Glycobiology* **16,** 71R–81R (2006).
